# Secondary Infections in Febrile Neutropenia in Hematological Malignancies: More Than Another Febrile Neutropenic Episode

**DOI:** 10.4274/tjh.2013.0422

**Published:** 2015-08-01

**Authors:** Aslıhan Demirel, Fehmi Tabak, M. Cem Ar, Bilgül Mete, Şeniz Öngören, Mücahit Yemişen, Reşat Özaras, Emre Eşkazan, Zafer Başlar, Ali Mert, Teoman Soysal, Burhan Ferhanoğlu, Yıldız Aydın, Recep Öztürk

**Affiliations:** 1 İstanbul University Cerrahpaşa Faculty of Medicine, Department of Infectious Diseases and Clinical Microbiology, İstanbul, Turkey; 2 İstanbul University Cerrahpaşa Faculty of Medicine, Department of Internal Medicine, Division of Hematology, İstanbul, Turkey

**Keywords:** Secondary infection, Febrile neutropenia, Hematological malignancy, mortality, Leukemia, Lymphoma

## Abstract

**Objective:**

Febrile neutropenic episodes (FNEs) are among the major causes of mortality in patients with hematological malignancies. Secondary infections develop either during the empirical antibiotic therapy or 1 week after cessation of therapy for a FNE. The aim of this study was to investigate the risk factors associated with secondary infections in febrile neutropenic patients.

**Materials and Methods:**

We retrospectively analyzed 750 FNEs in 473 patients between January 2000 and December 2006.

**Results:**

Secondary infections were diagnosed in 152 (20%) of 750 FNEs. The median time to develop secondary infection was 10 days (range: 2-34 days). The duration of neutropenia over 10 days significantly increased the risk of secondary infections (p<0.001). The proportion of patients with microbiologically documented infections was found to be higher in primary infections (271/750, 36%) compared to secondary infections (43/152, 28%) (p=0.038). Age; sex; underlying disease; antibacterial, antifungal, or antiviral prophylaxis; blood transfusion or bone marrow transplantation; central venous catheter; and severity of neutropenia did not differ significantly between primary and secondary infections (p>0.05). While fever of unknown origin (p=0.005) and catheter-related bacteremia (p<0.001) were less frequently observed in secondary infections, the frequency of microbiologically (p=0.003) and clinically (p<0.001) documented infections, fungal pneumonias (p<0.001), infections related to gram-positive bacteria (p=0.04) and fungi (p<0.001), and 30-day mortality rate (p<0.001) were significantly higher in cases of secondary infections (p<0.001).

**Conclusion:**

Secondary infections should be regarded as life-threatening complications of febrile neutropenia. Secondary infections represent a more severe and mortal complication and cannot be regarded just as another FNE.

## Abstract (tr)

**Amaç:**

Febril nötropenik atak (FNA) hematolojik hastalığı olan hastalarda ölümlerin başlıca nedenleri arasında yer almaktadır. İkincil enfeksiyonlar FNA’nın ampirik antibiyotik tedavi ya da bu tedavinin kesilmesinden bir hafta sonrasına kadar gelişebilir. Bu çalışmada amacımız, febril nötropenik hastalarda ikincil enfeksiyonları ile ilişkili risk faktörlerini araştırmaktır.

**Gereç ve Yöntemler:**

Kliniğimizde Ocak 2000 ile Aralık 2006 tarihleri arasında 73 hastada 750 FNA geriye dönük olarak incelenmiştir.

**Bulgular:**

Enfeksiyonlar 750 FNA’nın 152’sinde (%20) tespit edilmiştir. Sekonder enfeksiyon gelişmesi için geçen medyan süre 10 gün (1-34 gün) idi. Nötropeni süresinin 10 günün üzerinde olması ikincil enfeksiyon riskini anlamlı derecede arttırmaktadır (p<0,001). Mikrobiyolojik dökümante enfeksiyon oranı, primer enfeksiyonu (271/750, %36) olan hastalarda sekonder enfeksiyonlu hastalara (43/152, %28) göre daha yüksek olduğu bulunmuştur (p=0,038). Yaş, cinsiyet, altta yatan hastalık, antibakteriyel, antifungal veya antiviral profilaksi, kan transfüzyonu veya kemik iliği nakli, santral venöz kateter ya da nötropeni şiddeti açısından primer ve sekonder enfeksiyonu olan hastalar arasında anlamlı farklılık yoktu (p>0,05). Katetere bağlı bakteremi (p<0,001) ve nedeni bilinmeyen ateş (p=0,005) ikincil enfeksiyonlu hastalarda daha az sıklıkta gözlenirken, mikrobiyolojik (p=0,003) ve klinik olarak dökümante enfeksiyonlar (p<0,001), fungal pnömoniler (p<0,001), gram-pozitif bakteriler ile ilgili enfeksiyonlar (p=0,04) ve mantar enfeksiyonları (p<0,001) ve 30-günlük mortalite oranı (p<0,001) ikincil enfeksiyonlarda (p<0,001) anlamlı olarak yüksek bulunmuştur.

**Sonuç:**

İkincil enfeksiyonlar febril nötropenik hastada hayatı tehdit eden komplikasyonlar olarak kabul edilmelidir. Enfeksiyonlar daha ağır ve ölümcül şekilde ortaya çıkabilir ve yeni bir febril nötropenik atak olarak kabul edilmemelidir.

## INTRODUCTION

Infections are one of the most important causes of mortality in cancer patients [1]. More than 80% of patients receiving chemotherapy experience at least 1 febrile episode during neutropenia and 5%-10% of these patients are lost despite broad-spectrum antibiotherapy [2]. These infections should be treated effectively and rapidly. Previous studies showed that infections were the cause of death for 50%-80% of acute leukemia patients and for 50% of patients with lymphoma and solid tumors [3].

Infections that were not observed at the beginning and develop either during empirical antibiotic therapy or within 1 week after cessation of therapy in neutropenic patients are called ‘secondary infections’ or ‘superinfections’. A few studies suggested that secondary infections significantly increased mortality and that various factors may predict the development of secondary infections [4].

In our study, we aimed to investigate the frequency of secondary infections in febrile neutropenic patients, the risk factors for the development of secondary infections, and the differences between the febrile neutropenic patients developing and not developing secondary infections.

## MATERIALS AND METHODS

The analysis was conducted on a pooled database of febrile neutropenia in patients with hematological malignancies followed in a 1500-bed tertiary care center by a joint study group of the Hematology and Infectious Diseases Departments in the Cerrahpaşa Medical Faculty of İstanbul University.

A total of 750 febrile neutropenic episodes (FNEs) in 473 patients hospitalized between January 2000 and December 2006 were included in this retrospective study.

‘Fever’ was defined as a single oral temperature of ≥38.5 °C (axillary temperature of ≥38 °C) and absence of any other factors that may lead to fever or oral temperature of ≥38 °C (axillary temperature of ≥37.5 °C) for at least 2 successive measurements performed at 4-h intervals during a 24-h follow-up period. ‘Neutropenia’ was defined as absolute neutrophil count of ≤500/mm3 or 500-1000/mm3 but expected to fall below 500/mm3 within 24-48 h.

Only patients who responded to the initial therapy were included for analysis to clearly analyze the outcomes of primary and secondary FNE attacks. A secondary infection was defined as any episode of fever and/or infection not present at the initial evaluation that developed either during empirical therapy or within 1 week after discontinuation of therapy. The recurrence of fever in 48 h after its resolution during empirical antibiotic therapy was also considered as secondary infection [4].

FNEs were classified as microbiologically documented, clinically documented, or fever of unknown origin (FUO) if the cultures remained sterile and no diagnosis was identified on day 3. Fever related to any noninfectious cause (drug fever, activation of the underlying disease, transfusion-related) was considered ‘fever due to noninfectious causes’.

Secondary infections were also classified as microbiologically documented, clinically documented, or FUO. Minor fungal infections such as oral candidiasis were not assessed. The first 30 days of patients’ treatment were taken into consideration for analysis. The demographic data and underlying risk factors including severity and duration of neutropenia, core temperature, and C-reactive protein values were recorded for every patient on days 0, 3, 7, 14, and 30. Primary and secondary infections if detected, isolated pathogens, and the antibiotics used were assessed. Causes of any mortality after primary or secondary infections were investigated.

Secondary fungal infections were defined as infections with fungal agents starting during or 1 week after the cessation of an empirical antibiotic treatment [4]. Breakthrough infection was defined as the occurrence of an invasive fungal infection while the patient was receiving an effective antifungal drug as prophylaxis or therapy [5].

The chi-square test was used to compare statistically categorical variables and the Mann-Whitney U or t-test was used to compare continuous variables. A p-value of less than 0.05 was considered as statistically significant.

## RESULTS

A total of 750 primary infections were assessed. The median age of the patients was 39 years (range: 15-83 years), and there were 201 female (42.5%) and 272 male (57.5%) patients. The demographic characteristics of the patients are demonstrated in Table 1.

Prior prophylactic antibiotic use was recorded in 24% (185) of all FNEs. More than 1 prophylactic agent was used in 28 of these episodes. Central venous catheters (CVCs) were present in 169 (22.5%) of the patients at the time of episode.

Secondary infections were determined in 152 (20%) of 750 episodes. Secondary infections developed after 2 primary episodes in 13 patients and after 3 primary episodes in 1 patient. The median age of the patients developing secondary infections was 39 years (15-79 years).

The mean duration of neutropenia was 12 days (1-74 days). The absolute neutrophil count was <100/mm3 and ≥100/mm3 in 472 (62.1%) and 277 (36.4%) of the episodes, respectively.

The median time to development of secondary infection was 10 days (range: 2-34 days).

Secondary infections developed 48 h after the resolution of fever in 131 (86%) of the episodes while they developed within 1 week after the discontinuation of antibiotherapy in 21 (13%) episodes.

Factors related to secondary infection are outlined in Table 2.

A comparison of diagnoses in primary and secondary infections is given in Table 3.

Since all patients with bone marrow transplantation (BMT) received routine prophylaxis, we compared nontransplanted patients with or without antibacterial/antifungal prophylaxis in terms of secondary infections developments. We found that antibacterial and antifungal prophylaxis did not increase the risk of developing secondary infections (p=0.076) (Table 2).

We could not demonstrate any significant differences between nontransplanted and transplanted patients in term of secondary infections. In 152 episodes that led to the development of secondary infections, the neutrophil counts during the secondary infection were <100/mm3 for 87 (57.2%) episodes and ≥100/mm3 for 65 (42.8%) episodes (p=0.062) (Table 2).

The incidence of secondary infections varied significantly with respect to duration of neutropenia; duration of neutropenia was ≤10 days and >10 days in 7.16% and 34.1% of patients, respectively (p<0.001) (Figure 1). The respiratory system was considered to be the major site of infections in 86 primary and 53 secondary infections. Among these infections, the incidence of fungal pneumonia was found to be higher in secondary compared to primary infections (p<0.001).

Neither having a CVC nor the underlying disease was associated with increased risk for secondary infections at p=0.4 and p=0.09, respectively. Catheter-related bacteremia was more prevalent in primary (102 episodes) than in secondary (11 episodes) infections (p<0.001).

The etiologic agents detected in the primary and secondary infections are shown in Table 4. Gram-positive bacteria and fungi had significantly higher incidence rates in secondary compared to primary infections (p=0.04 and p<0.001). We did not detect any fungal breakthrough infection.

More than 1 pathogen was identified in 20 episodes during primary infection and in 5 episodes during secondary infection. Extended spectrum beta-lactamase-producing E. coli was identified in 3 of the primary episodes and 2 of the secondary infection episodes. Vancomycin-resistant Enterococcus sp. was isolated in 1 of the primary episodes.

Although the proportion of patients with microbiologically diagnosed infections was higher in primary infections (26%) than in secondary infections (19%), the difference was not significant (p=0.2).

The mortality rate of patients who developed secondary infections (22.7%) was significantly higher compared to patients who did not develop secondary infections (13.5%) (p<0.001).

## DISCUSSION

Various studies identify acute leukemia, lymphoma, and multiple myeloma as underlying diseases at rates of 53%-62%, 22%-27%, and 3%-14%, respectively, in febrile neutropenic patients [6,7,8]. In our study, similarly to other studies, acute leukemia, chronic leukemia, lymphoma, and multiple myeloma were determined in 51.1%, 7.3%, 24.9%, and 8.7% of 472 patients, respectively.

In recent studies, while gram-positive bacteria were identified at the rate of 44%-67%, gram-negative bacteria were determined at the rate of 55%-85% [8,9,10]. In our study, gram-positive and gram-negative bacteria isolated from various foci were almost evenly distributed at 49% and 48%, respectively. The use of intravascular catheters, antimicrobials such as ciprofloxacin or trimethoprim-sulfamethoxazole for prophylaxis, and the development of serious mucositis related with new chemotherapeutic agents are considered to underlie the increase in gram-positive bacteria [11,12].

The mortality rates were reported as 3%-8% in febrile neutropenia [7,8,13]. In our study, the mortality rate was 13.5% for the patients that had not developed any secondary infections. Mortality was linked to the uncontrolled, nonresponsive underlying disease in 33.9% of all deaths. Viscoli et al. estimated the mortality rate as 8%, and infection was identified as the cause in 30% of such events [8]. Since our tertiary care center is one of the biggest hospitals in the region, it is a referral center for nonresponsive and relatively late-stage patients. Intense chemotherapy programs applied to such patients may account for high mortality rates.

The incidence of secondary infections was 20% in our study, similar to what is reported in the literature, which varies between 12% and 24% [14,15]. It is interesting that all of these patients had acute leukemia or had undergone bone marrow transplantation. Nucci et al. examined 46 (14%) secondary infection episodes of 333 FNEs [16]. The latest study about secondary infections was carried out by the International Antimicrobial Therapy Group, a subgroup of the European Organization for Research and Treatment of Cancer (IATG/EORTC) [4]. In this study, secondary infections were reported in 129 (15%) of 836 patients responding to empirical treatment among 1720 patients with their first FNEs [4].

Serra et al. described the duration and severity of neutropenia, while Feld et al. described long-term antibacterial therapy and lack of response to empirical treatment as significant risk factors for the development of secondary infections [14,15]. In the study performed by Akova et al., adult age, acute leukemia in the first induction phase, presence of intravascular catheter, absolute neutrophil count of <100/mm3 on day 4, and the identification of any other cause of the secondary infection apart from the clinically documented infection significantly increased the risk of developing secondary infections [4].

In our study, we found that duration of neutropenia of over 10 days significantly increased the risk for secondary infection development. A significant relationship was not shown between secondary infection development and age, sex, underlying disease, severity of neutropenia, use of catheter, or administration of prophylaxis.

The incidence and the severity of infections were related with the severity and duration of neutropenia. In most studies, a significant relationship was determined between the duration and severity of neutropenia and the development of secondary infections [4,14,15,16]. Pizzo et al. reported a strong correlation between the duration of neutropenia and superinfection (secondary infection). While superinfection was not observed in patients with neutropenia lasting less than 7 days, it was seen in 47% of patients with neutropenia of longer duration [17]. The frequency of secondary infections in our study was 7.16% and 34.1% in patients with neutropenia lasting ≤10 days and >10 days, respectively.

We isolated fungal pathogens more frequently in cases of secondary infections compared to primary infections (p<0.001). Furthermore, the fungal infections documented clinically and radiologically rather than microbiologically were identified with a significantly higher rate in secondary infections compared to primary ones (p<0.001). This may be explained by the fact that prolonged neutropenia is the most important risk factor for fungal infections [18]. However, when secondary infections were examined discretely, fungal pathogens, gram-negative bacteria, and gram-positive bacteria were observed at rates of 11%, 37%, and 32%, respectively. Fungal agents accounted for 67% of the secondary infections in the study by Nucci et al., 48% in the study by Akova et al., and 24.7% in the study by Serra et al. [4,14,16]. The severity of the clinical picture of the patients included in our study and the difficulty of obtaining samples for culture due to the lack of adequate platelet counts in most of these patients may account for this difference.

Since patients undergoing BMT receive antibiotic prophylaxis on a routine basis, we excluded these patients when assessing the impact of prophylaxis on the development of secondary infections. No significant relationship was determined between prophylaxis and development of secondary infections. Similarly, Serra et al. did not find any relationship between prophylaxis and development of secondary infections [14]. In the study by Akova et al., an increased rate of gram-positive infections was associated with the administration of antibacterial prophylaxis [4]. On the other hand, Nucci et al. reported that omitting quinolone prophylaxis resulted in a higher risk of secondary infections [16]. The relatively lower frequency of gram-positive bacteria may be explained by the use of quinolone prophylaxis in our study.

In the study carried out by Akova et al., it was reported that the median time to develop secondary infections was approximately 10 days (range: 1-28 days) [4]. Similarly, the median time was 10 days (range: 2-34 days) in our study. Secondary infections developed under antibiotherapy in 131 of the episodes, whereas 22 episodes developed within 1 week after the discontinuation of antibiotherapy.

In a study by Serra et al., microbiologically documented infections accounted for 39.8% of primary episodes and 90.1% of secondary infections [14]. In our study, the rate of microbiologically documented infections in primary infections was significantly higher compared to secondary infections (p=0.003). Collection of cultures before the administration of antibiotherapy could have facilitated the identification of the agents in primary infections. The rate of clinically documented infections was significantly higher among secondary infections than primary infections (p<0.001). This may be explained by the higher rate of fungal infections. The inability to perform interventions for most patients due to severe thrombocytopenia further complicates microbiological documentation.

The presence of CVCs predisposes to a risk for development of bacteremia and fungemia [19]. The catheter-related bacteremia rate was high in primary episodes in our study and methicillin-resistant coagulase-negative staphylococci (MRCNS) were identified as the most common cause. Considering that the most common agents identified in the catheter-related bacteremia cases were MRCNS, S. aureus, aerobic gram-negative bacilli, and C. albicans, our finding was consistent with the literature [20]. The frequency of catheter-related bacteremia in primary infections was higher than in secondary infections.

In some of the earlier studies on secondary infections, it was reported that the use of intravenous catheters increased the risk of developing secondary infections [4,16]. However, in our study, no significant relationship was determined, similarly to some other studies [14,15].

There are several important prognostic factors increasing mortality in febrile neutropenic patients [1]. In our study, fungal infections accounted for 33% of the cases with secondary infections and a mortal course. In the study carried out by Nucci et al., fungal infections were responsible for 55% of the mortality [16]. Consistent with that, this study also shows that the development of secondary infections in these patients is an important factor increasing mortality (p<0.001).

To conclude, secondary infections were considered as common and life-threatening complications of febrile neutropenia. Duration of neutropenia over 10 days significantly increased the secondary infection risk. Fungal infections were more frequently seen in secondary infections compared to primary infections. This was considered to be related to the long duration of neutropenia and long-term use of broad-spectrum antibiotics.

Mortality was also significantly increased in patients with secondary infections. Thus, secondary infections should be considered as a complicated, more mortal form of FNE with a differing clinical and etiological spectrum.

## Figures and Tables

**Table 1:**
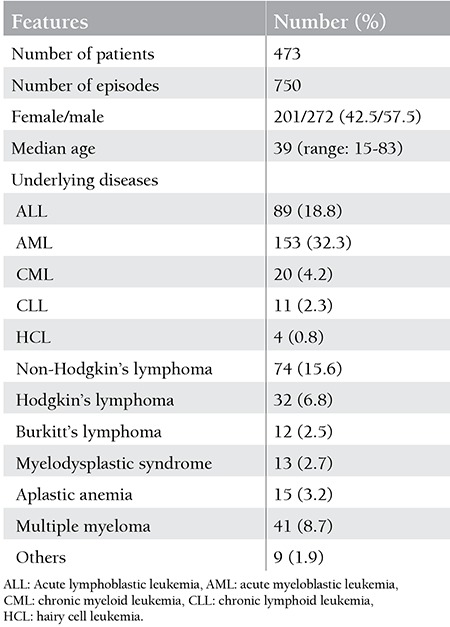
Demographic characteristics of the cases with primary febrile neutropenic episodes.

**Table 2:**
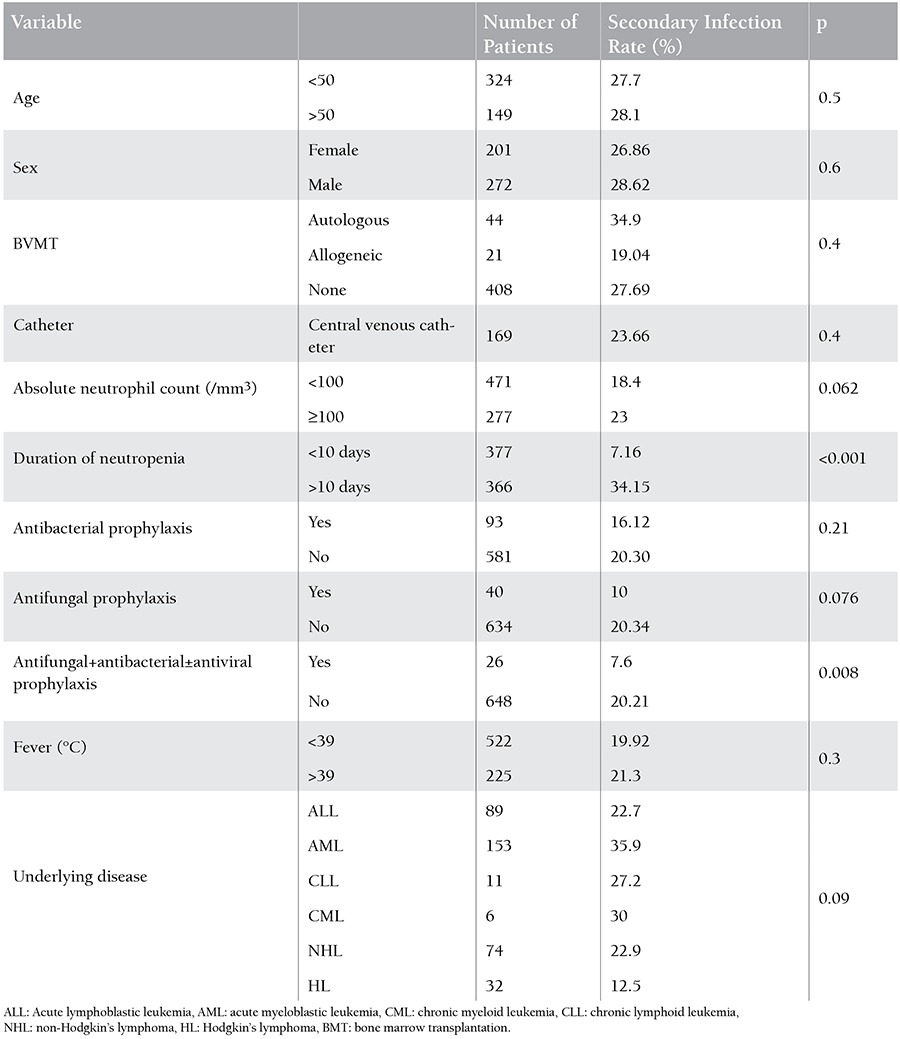
Factors related to secondary infection developed in out of 750 primary febrile neutropenic episodes.

**Table 3:**
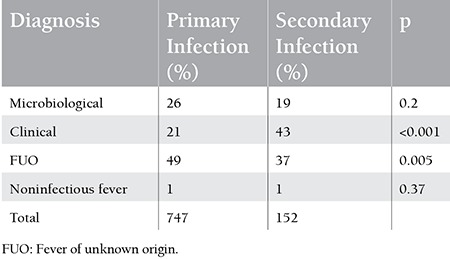
Comparison of diagnoses in the primary and secondary infections.

**Table 4:**
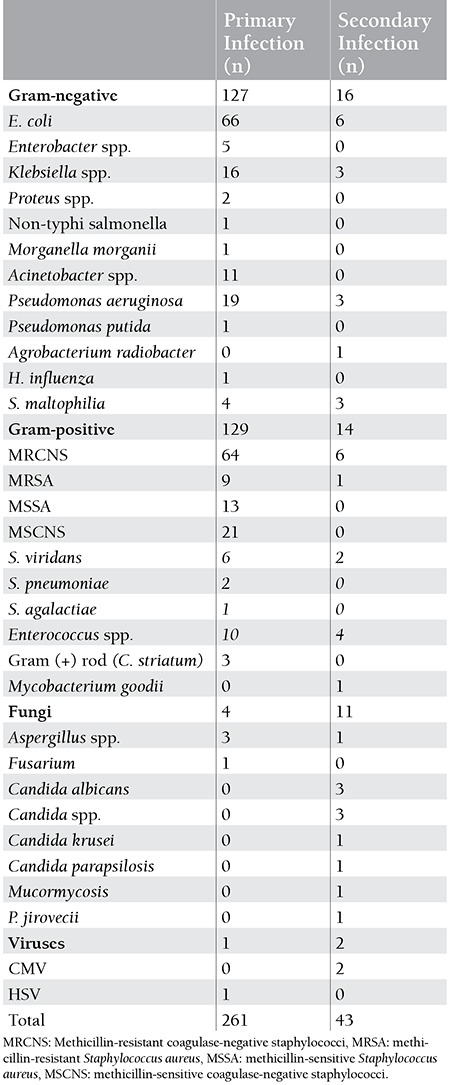
Distribution of etiological agents in the primary and secondary infections.

## References

[1] González-Barca E, Fernández-Sevilla A, Carratalá J, Salar A, Peris J, Grañena A, Gudiol F (1999). Prognostic factors influencing mortality in cancer patients with neutropenia and bacteremia. Eur J Clin Microbiol Infect Dis.

[2] Glasmacher A, von Lilienfeld-Toal M, Schulte S, Hahn C, Schmidt-Wolf IG, Prentice A (2005). An evidence-based evaluation of important aspects of empirical antibiotic therapy in febrile neutropenic patients. Clin Microbiol Infect.

[3] Viscoli C (1998). The evolution of the empirical management of fever and neutropenia in cancer patients. J Antimicrob Chemother.

[4] Akova M, Paesmans M, Calandra T (2005). A European Organization for Research and Treatment of Cancer-International Antimicrobial Therapy Group Study of secondary infections in febrile, neutropenic patients with cancer. Clin Infect Dis.

[5] Nailor MD, Chandrasekar PH (2009). Treatment of breakthrough fungal infections: Is there one best drug strategy?. Current Fungal Infection Reports.

[6] Cherif H, Björkholm M, Engervall P, Johansson P, Ljungman P, Hast R, Kalin M (2004). A prospective, randomized study comparing cefepime and imipenem-cilastatin in the empirical treatment of febrile neutropenia in patients treated for haematological malignancies. Scand J Infect Dis.

[7] Rossini F, Terruzzi E, Verga L, Larocca A, Marinoni S, Miccolis I, Giltri G, Isella M, Parma M, Pogliani EM (2005). A randomized clinical trial of ceftriaxone and amikacin versus piperacillin tazobactam and amikacin in febrile patients with hematological neoplasia and severe neutropenia. Support Care Cancer.

[8] Viscoli C, Cometta A, Kern WV, Bock R, Paesmans M, Crokaert F, Glauser MP (2006). Piperacillin-tazobactam monotherapy in high-risk febrile and neutropenic cancer patients. Clin Microbiol Infect.

[9] Cordonnier C, Buzyn A, Leverger G, Herbrecht R, Hunault M, Leclercq R, Bastuji-Garin S;, Club de Réflexion sur les Infections en Onco-Hématologie (2003). Epidemiology and risk factors for gram-positive coccal infections in neutropenia: toward a more targeted antibiotic strategy. Clin Infect Dis.

[10] Winston DJ, Lazarus HM, Beveridge RA, Hathorn JW, Gucalp R, Ramphal R, Chow AW, Ho WG, Horn R, Feld R, Louie TJ, Territo MC, Blumer JL, Tack KJ (2001). Randomized, double-blind, multicenter trial comparing clinafloxacin with imipenem as empirical monotherapy for febrile granulocytopenic patients. Clin Infect Dis.

[11] Ramphal R (2004). Changes in the etiology of bacteremia in febrile neutropenic patients and the susceptibilities of the currently isolated pathogens. Clin Infect Dis.

[12] Zinner SH (1999). Changing epidemiology of infections in patients with neutropenia and cancer: emphasis on gram-positive and resistant bacteria. Clin Infect Dis.

[13] Bow EJ, Rotstein C, Noskin GA, Laverdiere M, Schwarer AP, Segal BH, Seymour JF, Szer J, Sanche S (2006). A randomized, open-label, multicenter comparative study of the efficacy and safety of piperacillin-tazobactam and cefepime for the empirical treatment of febrile neutropenic episodes in patients with hematologic malignancies. Clin Infect Dis.

[14] Serra P, Santini C, Venditti M, Mandelli F, Martino P (1985). Superinfections during antimicrobial treatment with betalactam-aminoglycoside combinations in neutropenic patients with hematologic malignancies. Infection.

[15] Feld R, Goodman PJ, Higgins B, Prognostic factors for the development of superinfections in febrile neutropenic cancer patients (abstract 1695) (1992). In: Program and Abstracts of the 32nd Interscience Conference on Antimicrobial Agents and Chemotherapy. DC.

[16] Nucci M, Spector N, Bueno AP, Solza C, Perecmanis T, Bacha PC, Pulcheri W (1997). Risk factors and attributable mortality associated with superinfections in neutropenic patients with cancer. Clin Infect Dis.

[17] Pizzo PA, Ladisch S, Ribichaud K (1980). Treatment of gram-positive septicemia in cancer patients. Cancer.

[18] Bodey GP (1998). Fungal infections in cancer patients. Ann N Y Acad Sci.

[19] Raad II, Bodey GP (1992). Infectious complications of indwelling vascular catheters. Clin Infect Dis.

[20] Maki DG, Mermel LA, In: Bennett JV, Brachman PS (1998). Infections due to infusion therapy. Hospital Infections.

